# Fine mapping of an up-curling leaf locus (*BnUC1*) in *Brassica napus*

**DOI:** 10.1186/s12870-019-1938-0

**Published:** 2019-07-19

**Authors:** Mao Yang, Chengwei Huang, Mingming Wang, Hao Fan, Shubei Wan, Yangming Wang, Jianbo He, Rongzhan Guan

**Affiliations:** National Key Laboratory of Crop Genetics and Germplasm Enhancement, Jiangsu Collaborative Innovation Center for Modern Crop Production, Nanjing Agricultural University, Nanjing, 210095 China

**Keywords:** *Brassica napus*, Up-curling leaf mutant, Single nucleotide polymorphism, Near-isogenic line, Gene mapping

## Abstract

**Background:**

Leaf shape development research is important because leaf shapes such as moderate curling can help to improve light energy utilization efficiency. Leaf growth and development includes initiation of the leaf primordia and polar differentiation of the proximal-distal, adaxial-abaxial, and centrolateral axes. Changes in leaf adaxial-abaxial polarity formation, auxin synthesis and signaling pathways, and development of sclerenchyma and cuticle can cause abnormal leaf shapes such as up-curling leaf. Although many genes related to leaf shape development have been reported, the detailed mechanism of leaf development is still unclear. Here, we report an up-curling leaf mutant plant from our *Brassica napus* germplasm. We studied its inheritance, mapped the up-curling leaf locus *BnUC1*, built near-isogenic lines for the *Bnuc1* mutant, and evaluated the effect of the dominant leaf curl locus on leaf photosynthetic efficiency and agronomic traits.

**Results:**

The up-curling trait was controlled by one dominant locus in a progeny population derived from NJAU5734 and Zhongshuang 11 (ZS11). This *BnUC1* locus was mapped in an interval of 2732.549 kb on the A05 chromosome of *B. napus* using Illumina Brassica 60 K Bead Chip Array. To fine map *BnUC1*, we designed 201 simple sequence repeat (SSR) primers covering the mapping interval. Among them, 16 polymorphic primers that narrowed the mapping interval to 54.8 kb were detected using a BC_6_F_2_ family population with 654 individuals. We found six annotated genes in the mapping interval using the *B. napus* reference genome, including BnaA05g18250D and BnaA05g18290D, which bioinformatics and gene expression analyses predicted may be responsible for leaf up-curling. The up-curling leaf trait had negative effects on the agronomic traits of 30 randomly selected individuals from the BC_6_F_2_ population. The near-isogenic line of the up-curling leaf (ZS11-UC1) was constructed to evaluate the effect of *BnUC1* on photosynthetic efficiency. The results indicated that the up-curling leaf trait locus was beneficial to improve the photosynthetic efficiency.

**Conclusions:**

An up-curling leaf mutant *Bnuc1* was controlled by one dominant locus *BnUC1*. This locus had positive effects on photosynthetic efficiency, negative effects on some agronomic traits, and may help to increase planting density in *B. napus*.

**Electronic supplementary material:**

The online version of this article (10.1186/s12870-019-1938-0) contains supplementary material, which is available to authorized users.

## Background

Leaves are the primary photosynthetic organs in most plants. Leaf flat and curling have a direct impact on light absorption and transmittance. Changes in leaf morphology can enhance the overall light energy utilization efficiency and improve plant yields. Therefore, research on genes controlling leaf shape is important for plant breeding and improvement, as well as for plant developmental biology.

Leaf formation is regulated by complex developmental processes including pattern formation, polarity establishment, and cell differentiation [1–5]. The morphology of mature leaves is synergistically accomplished by the development of the proximal-distal, adaxial-abaxial, and centrolateral axes [6–9]. The adaxial-abaxial polarity affects and determines the development of the other two axes and, therefore, is crucial to curling leaf formation [10]. Alteration of the adaxial-abaxial polarity generally results in inward (adaxial) or outward (abaxial) leaf curling. Genes involved in establishing adaxial-abaxial polarity in leaves include those that encode homeodomain–leucine zipper (HD–ZIP) class III transcription factors [11], KANADI-type transcription factors [12, 13], and YABBY transcription factors [14, 15].

HD–ZIP III may affect the establishment of adaxial axis polarity. HD–ZIP III *Arabidopsis thaliana* mutants PHABULOSA (*phb*) [16], PHAVOLUTA (*phv*) [16], and REVOLUTA (*rev*) [17, 18] displayed an adaxial development phenotype caused by a mutation in the conserved START domain of HD–ZIP III, which contains binding sites for the microRNA165/166 [19–21]. Other HD–ZIP III genes were found to be expressed mainly in shoot apical meristem and adaxial axis of leaf primordium and to participate in adaxial axis development in cucumber [22], maize [23–25], and rice [16, 17, 19, 26, 27]. Functional deletions in conserved domains of these genes led to abnormal apical meristem and curling leaves with an enlarged abaxial axis [12].

KANADI transcription factor genes from the ARR-B and GOLDEN2-LIKE gene families, which are expressed mainly in the abaxial axis, antagonize the transcription of HD–ZIP III genes [28, 29]. A single mutation in *KAN1* or *KAN2* had little or no effect on leaf morphology, but a *kan1kan2* double mutation led to the replacement of abaxial cell types with adaxial cell types in most lateral organs, leading to curling leaves [12]. In rice, the KANADI transcription factor SHALLOT-LIKE1 was found to be involved in the establishment of abaxial cell polarity [30]. YABBY transcription factor genes also may be abaxial axis determinants downstream of KANADI genes [1, 14]. In *A.thaliana*, members of the YABBY gene family, including *FIL, INO, CRC, YAB2, YAB3*, and *YAB5,* are expressed mainly in the abaxial surface of lateral organs [1, 14, 31–33]. The YAB domain of *FIL* and *YAB3* regulates abaxial patterning, growth of lateral organs, and inflorescence phyllotaxy [34]. Loss of function of YABBY genes leads to loss of leaf polarity.

Besides the genes mentioned above, other genes related to leaf polarity formation have been found. For example, ASYMMERTIC LEAVES1 (*AS1*) and *AS2* promote the expression of HD–ZIP III genes in the adaxial axis by inhibiting the transcription of miR165/166 [35]. In leaf primordia, *AS1* regulates the adaxial-abaxial axis as a determinant of leaf curl, whereas *AS2* inhibits the transcription of KANADI and YABBY genes. KANADI genes can also negatively regulate the expression of YABBY genes by binding to the promoter region of *AS2* [36]. The first gene identified for adaxial-abaxial leaf differentiation, *AS1* (also known as *PHAN*), was cloned from snapdragon [37]. The multilayer control network of adaxial-abaxial polarity implies numerous genes are involved in controlling the normal development of leaf.

Plant endogenous hormones also are related to leaf curl [38]. Mutations or expression changes in genes involved in plant hormone biosynthesis or signaling pathways have been found to change leaf morphology, including leaf curl [9, 39–42]. Such changes in leaf morphology are usually accompanied by whole plant changes, but the corresponding mechanism for whole plant development is not discussed here.

Here, we report a dominant up-curling leaf mutant (*Bnuc1*) discovered from our pure *Brassica napus* line, NJAU5734. We studied the inheritance of the up-curling leaf locus (*BnUC1*), built near-isogenic lines (NILs) for *Bnuc1*, and mapped the up-curling leaf locus. Further, we evaluated the effect of the dominant leaf curl locus on leaf chlorophyll content, photosynthetic efficiency, and agronomic traits. Our results will lay a good foundation for elucidating the molecular mechanism underlying the up-curling leaf phenotype in *B. napus*, and provide clues for further positional cloning and functional research related to *BnUC1*.

## Results

### Performance of the up-curling leaf mutant

**Fig. 1:**
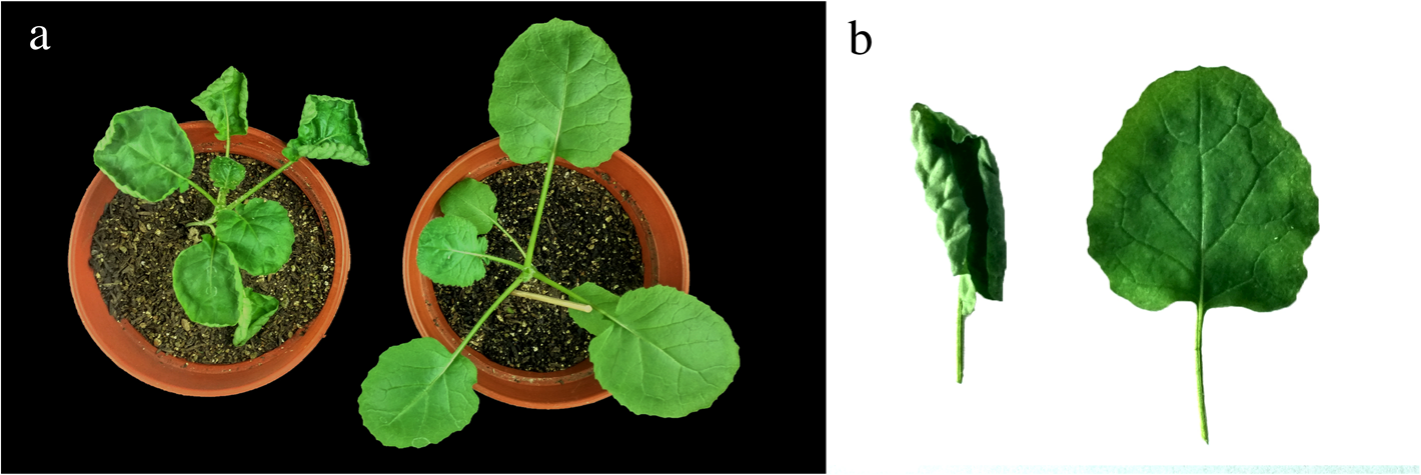
Leaf morphology of the parents used for the up-curling leaf trait inheritance studies. **a** and **b** show the leaves of the parent NJAU5734 with up-curling leaves (left) and the parent ZS11 with flat leaves (right) at the seeding stage

The parent NJAU5734 has an up-curling leaf phenotype at the seedling stage and during the leaf development period, whereas the canola parent ZS11 has normal flat leaves (Fig. 1). After the bolting stage, the leaves of NJAU5734 were up-curling along the middle axis and became upright. The NIL ZS11-UC1 with the genetic background of ZS11 has an up-curling leaf phenotype that is similar to the leaf phenotype of NJAU5734 (Additional file 1: Figure S1).

The leaf Chl a, Chl b, and total Chl content and the Chl a to Chl b ratio in ZS11-UC1 were significantly higher than those in ZS11 (Table 1). This result indicates that the up-curling leaf trait is associated with elevated leaf Chl content.

**Table 1:** Leaf chlorophyll contents of the ZS11 and its near isogenic line ZS11-UC1 at up-curling leaf locus

Genotype	Chl a (mg/g)	Chl b (mg/g)	Total	Chl a/b ratio
ZS11	4.18 ± 0.54	2.73 ± 0.27	6.59 ± 0.81	1.67 ± 0.20
ZS11-UC1	6.70 ± 0.62^a^	3.54 ± 0.23^a^	10.24 ± 0.95^a^	1.89 ± 0.22^a^

^a^indicates significant at the 0.05 probability level. Mean ± standard deviation (*SD*) under sample size

The leaf net photosynthetic rate and stomatal conductance of ZS11-UC1 were significantly higher than those of ZS11, the leaf concentration of intercellular CO_2_ was significantly lower than in ZS11, and there was no significant difference in the leaf transpiration rate between the two NILs (Table 2). This result indicates the up-curling leaf trait is associated with elevated photosynthetic efficiency.

**Table 2:** Leaf photosynthetic indicators of the ZS11 and its near isogenic line ZS11-UC1 at up-curling leaf locus

Genotype	NPR *μ*mol CO_2_ m^−2^ s^−1^	SC mol H_2_O m^− 2^ s^− 1^	ICC μmol CO_2_ mol^− 1^	TR *m*mol H_2_O m^− 2^ s^− 1^
ZS11	8.68 ± 0.40	0.18 ± 0.02	461.00 ± 5.29	2.70 ± 0.41
ZS11-UC1	12.55 ± 0.89^a^	0.22 ± 0.02^a^	397.50 ± 6.41^a^	3.06 ± 0.38

Data are presented as means ± *SD, n = 6*. ^a^indicates significant at 0.01 probability level. NPR, SC, ICC and TR denotes net photosynthetic rate, stomatal conductance, intercellular CO_2_ concentration and transpiration rate, respectively

### Inheritance of the up-curling leaf trait

The F_1_ (ZS11 × NJAU5734) and RF_1_ (NJAU5734 × ZS11) plants obtained by crossing NJAU5734 (up-curling leaves) and ZS11 (normal flat leaves) all had the up-curling leaves, indicating the up-curling leaf trait was controlled by dominant genes. By selfing the F_1_ plants, we obtained a F_2_ population with 328 plants; 241 had up-curling leaves and 87 had flat leaves (Table 3). The Chi-square test showed that the segregation pattern of the leaf trait obeyed the expected Mendelian segregation ratio of 3:1 (up-curling leaf vs. flat leaf). Further, the backcross population derived from the two parents had 279 plants; 147 had up-curling leaves and 132 had flat leaves. This result agrees with the Mendelian inheritance ratio of 1:1 (up-curling leaf vs. flat leaf). Thus, we inferred the up-curling leaf trait may be controlled by a dominant locus. In subsequent backcrossing populations, for example the BC_6_F_2_ population, the genetic regulation was confirmed.

**Table 3:** Inheritance of the up-curling leaf trait in populations derived from the two parents in *B. napus*

Population	Up-curling	Flat	Total	Expectation	*χ* ^2^	*P* value
F_1_	30	0	30			
RF_1_	30	0	30			
F_2_	241	87	328	3:1	0.33	0.52
BC_1_	147	132	279	1:1	0.70	0.40
BC_6_F_2_	484	170	654	3:1	0.15	0.70

### Gene mapping

**Fig. 2:**
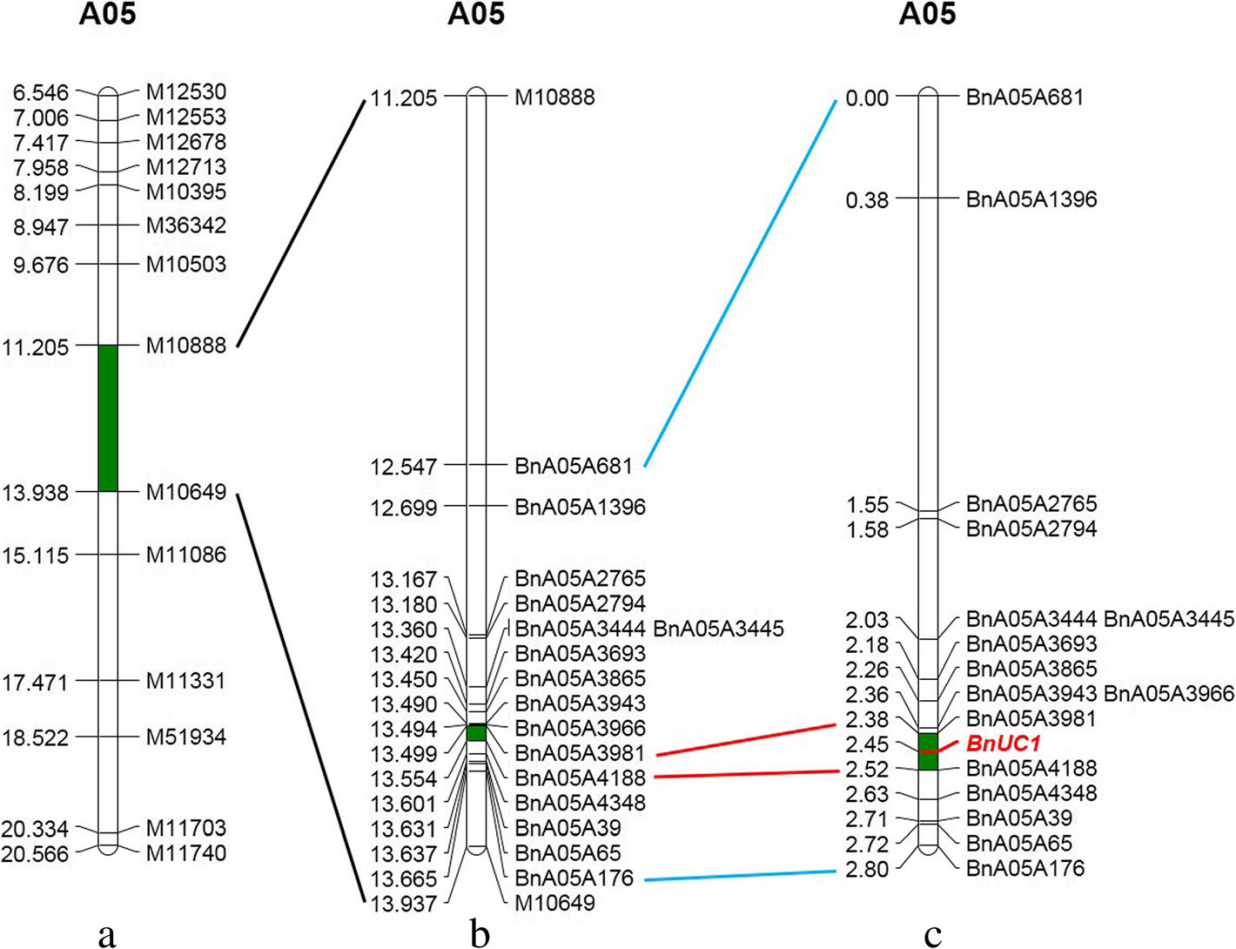
Mapping the up-curling locus *BnUC1*. **a** indicate the *BnUC1* locus was blue-colored in an interval of 2732.549-kb between SNP markers M10888 and M10649 on A05 chromosome. **b** shows that the *BnUC1* locus was fined mapped in the physical interval of 54.8 kb with facilitation of the developed SSR markers. **c** shows that *BnUC1* was in a 0.137 cM region between SSR markers BnA05A3981 and BnA05A4188

Twenty-two up-curling plants from the BC_6_ family population together with the parents NJAU5734 and the recurrent parent ZS11 were identified using the obtained SNP data on the 19 *B. napus* chromosomes. The 22 individuals contained only one common segment from the recurrent parent ZS11 on the A05 chromosome (Fig. 2a), in which 232 polymorphic SNP markers covering an interval of 2732.549 kb between SNP markers M10649 and M10888 were found to be co-segregated in the BC_6_ population. This indicated that this shared segment may harbor the dominant up-curling leaf locus. Among these 22 identified plants from the BC_6_ family population, a plant numbered ZS11-UC-6 was found to have only one region that was different from the corresponding region of the recurrent parent ZS11. Thus, this plant was selected and selfed to generate a BC_6_F_2_ family population for fine mapping the up-curling leaf locus.

**Fig. 3:**
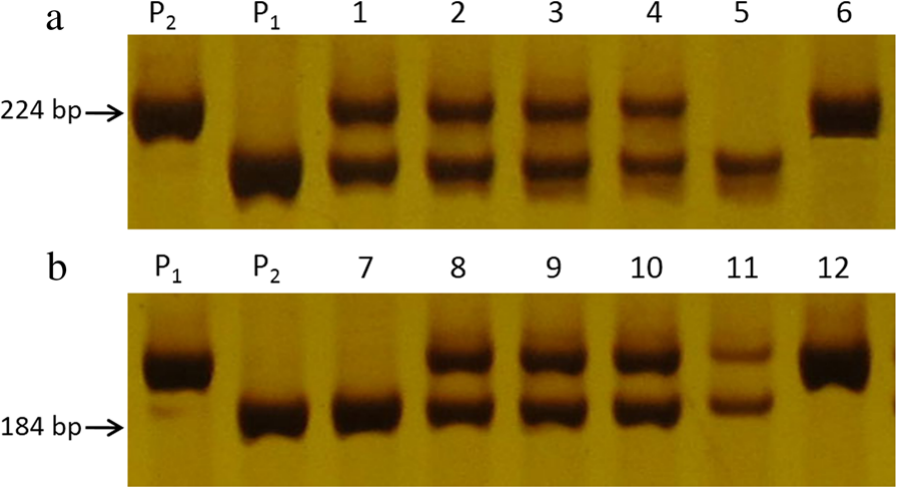
Partial molecular marker experimental results for co-dominant SSR markers BnA05A3981 and BnA05A4188. Marker scan with progeny BC_6_F_2_ family populations derived from the parents ZS11 and NJAU5734 was conducted. P_1_ and P_2_ indicates PCR products from the parents ZS11 and NJAU5734 plants, respectively. **a** shows partial results for SSR marker BnA05A3981 in which 1–4 denote the PCR products from the heterozygous plants in the progeny populations, and 5 and 6 denotes products from the homozygous plants with flat leaves and up-curling leaves, respectively. **b** shows partial results for SSR marker BnA05A3981 in which 8–11 denote the PCR products from the heterozygous plants in the progeny populations, and 7 and 12 denotes products from the homozygous plants with flat leaves and up-curling leaves, respectively

To fine map the *BnUC1* locus, locus-targeted genomic sequences were found by probing the genome with SNP markers. Based on the target sequence, 60 primer pairs of SSR markers were designed to uniformly cover the preliminary mapping interval. As a result, five polymorphic markers (BnA05A681, BnA05A1396, BnA05A2765, BnA05A2794, and BnA05A176) were detected in the mapping interval (Fig. 2b, c). The 654 individuals in the BC_6_F_2_ population were analyzed using these five SSR markers. The linkage map constructed using the SSR data and corresponding leaf shape phenotypes showed that these SSR markers were tightly linked with the *BnUC1* locus. The marker arrangements and recombination rate data agreed well with the physical genome map of *B. napus*, which indicates that the preliminary mapping was reliable. Using these results, the *BnUC1* locus was delimited to an interval of 485 kb between SSR markers BnA05A2794 and BnA05A176. Next, we designed 51 SSR primers within the new narrow mapping interval and detected six that were polymorphic (BnA05A3444, BnA05A3445, BnA05A3693, BnA05A3865, BnA05A39, and BnA05A65). These new makers helped to narrow the interval to 179 kb. Then, 90 SSR primers were designed within the 179 kb region, and five of them were found to be polymorphic (BnA05A3943, BnA05A3966, BnA05A3981, BnA05A4188, and BnA05A4348) (Fig. 2b, c). The polymorphic markers BnA05A3981 and BnA05A4188 formed clear bands and acted as co-dominant markers (Fig. 3). Finally, the *BnUC1* locus was mapped to a 54.8-kb interval between BnA05A3981 and BnA05A4188. No other markers to further narrow the mapping interval were found for this mapping population and its parents.

### Candidate gene analysis

Homologous segments of the mapping interval carrying the *BnUC1* locus were analyzed to detect candidate genes. Two *Brassica* genome databases were searched using SNP probes and SSR sequences, and two homologous A05 chromosome segments from *B. rapa* (http://brassicadb.org/brad/) and *B. napus* cv*.* ‘Darmor-*bzh*’ (http://www.genoscope.cns.fr/brassicanapus/) were detected (Table 4). The two homologous segments were aligned using BLASTN (http://blast.ncbi. nlm.nih.gov/Blast.cgi), which revealed the two segments shared 99% similarity. The two segments from *B. rapa* and *B. napus* were almost the same length, contained the same set of genes, and the annotated genes had relatively conserved protein coding sequences (Table 4).

**Table 4:** Genes on the mapped segments and their homologous *Brassica* segments on the A05 chromosome

Gene in *B. napus*	Gene in *B. rapa*	Homologue in *A. thaliana*	Gene function
BnaA05g18240D	Bra034420	AT1G35470.2	SPla/RYanodine receptor (*SPRY*) domain- containing protein
BnaA05g18250D	Bra034421	AT1G35460.1	*bHLH* transcript factor
BnaA05g18260D	Bra034422		unknown protein
BnaA05g18270D	Bra034423	AT3G56310.1	Melibiase family protein
BnaA05g18280D	Bra034424	AT1G35210.1	unknown protein
BnaA05g18290D	Bra034425	AT1G35190.1	2-oxoglutarate (2OG) and Fe(II)-dependent oxygenase superfamily protein

BnaA05g18250D, which was in the mapping interval, encodes a bHLH transcription factor and is homologous to a gene in The Arabidopsis Information Resource (TAIR) database, namely AT1G35460.1, the AtCFL1 associated protein (*CFLAP2*). CFLAP2 interacts with the C-terminal of CFL1 to regulate the downstream genes BODYGUARD (*BDG*) and FIDDLEHEAD (*FDH*), and negatively regulate cuticle development. When *CFL1* or *CFLAP2* is overexpressed, the expression of *BDG* and *FDH* are negatively regulated to decrease cuticle density, leading to defective cuticle development, which affects the differentiation of epidermal cells and changes the status of epidermal cells [43]. In the curly flag leaf 1 rice mutant, overexpression of *CFL1* down-regulated the expression of *BDG* and *FDH*, which affected the development of leaf cuticle and led to the curly flag leaf phenotype [44]. We infered that BnaA05g18250D (*BnCFLAP2*) also negatively regulates the expression of *BDG* and *FDH*, resulting in a defective leaf cuticle, which results in leaf up-curling. Based on these previous reports, BnA05g18250D may be our candidate gene.

BnaA05g18290D in the mapping interval is homologous to AT1G35190.1, a member of the 2-oxoglutarate- Fe (II)-dependent oxygenase superfamily to which the rice Rolling-leaf 14 (*RL14*) gene also belongs. RL14 has been reported to control the up-curling leaf trait in rice [45]. RL14 affects the formation of the secondary walls of thick-walled cells and mesophyll cells, which in turn affects water transport from vascular bundles of leaves to alveolar cells and the contraction of alveolar cells, resulting in leaves rolling [45]. BnaA05g18290D may have a similar function as *RL14* and be responsible for the leaf up-curling.

BnaA05g18240D in the mapping interval, is homologous to AT1G35470.2, which belongs to the SPRY (SPla and RYanodine receptor) domain-containing gene family. The conserved SPRY domain was identified originally as a structural motif in ryanodine receptors [46]. Later, proteins with SPRY domains were found to be involved in intracellular calcium release [47]. SPRY domain-containing proteins have not been reported to be related to leaf shape development.

BnaA05g18270D in the mapping interval, is homologous to AT3G56310.1, which encodes a member of the glycoside hydrolase family 27 that is an α-galactosidase (AGAL3). AGAL3 has a signal peptide at the N-terminus and is likely to be responsible for the hydrolysis of the β-l-arabinopyranoside residues in *A. thaliana* [48]. AGAL3 also has not been reported to be related to leaf shaping.

BnaA05g18260D and BnaA05g18280D in the mapping interval encode proteins of unknown function, and cannot be excluded from the list of candidate genes.

### Quantitative RT-PCR

We have analyzed the melting curve and amplification curves for each gene. The melting curve showed that each primers couple were specific (Additional file 2: Figure S2). The results showed that each primers can use to analysis gene expression. The amplification curves of eight genes and housekeep genes in the standard solution generated well standard curves for each qRT-PCR primers couple (Additional file 3: Figure S3). The results showed that the amplification curves of eight genes were parallel to housekeep genes. It showed that the amplification efficiency of eight genes and housekeep genes in the same DNA sample was the same (Additional file 4: Table S1). Thus, the qRT-PCR reaction system was qualified and could be used for subsequent population qRT-PCR experiments.

**Fig. 4:**
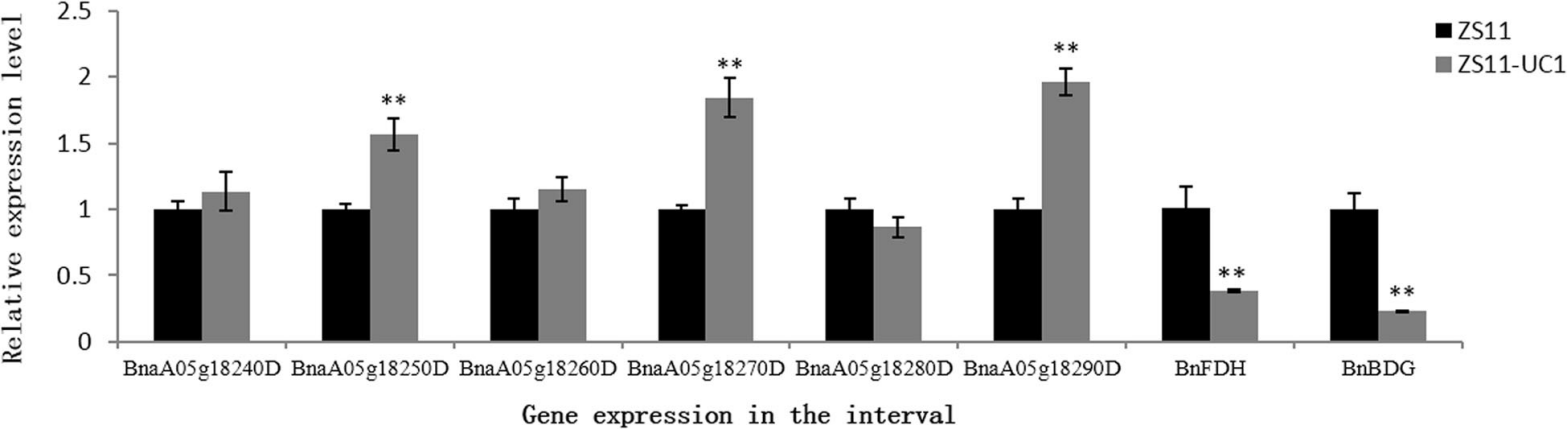
Differential expression of 6 genes in the mapping interval in leaves of the pair of near isogenic lines. Canola parent ZS11 and its near isogenic line ZS11-UC1 with up-curling leaves were used for gene expression experiment. The Actin of *B. napus* was used as the reference gene for normalization in qRT-PCR experiments. The relative mRNA level of genes in ZS11-UC1 shown in grey box was calculated in reference to gene expression level of ZS11. Values shown are means ± *SD* (*n* = 4). ** denotes significant at probability level of 0.01

The six genes detected in the mapping interval were quantified by qRT- PCR (Fig. 4). The results showed that the expression levels of three genes (BnaA05g18250D, BnaA05g18270D, and BnaA05g18290D) were significantly higher in the ZS11-UC1 mutant than in ZS11. There were no significant differences in the expression of the other three genes between the ZS11-UC1 mutant and ZS11. This result further confirms that BnaA05g18250D and BnaA05g18290D are good candidate genes for *BnUC1.* The differential expression of BnaA05g18270D between the two NILs may be because the two other differentially expressed genes disturb the gene regulation network. The three genes that showed no significant expression differences between the NILs were removed from the list of candidate genes.

To determine whether BnaA05g18250D (*BnCFLAP2*) can control the up-curling leaf phenotype, we determined the expression of two downstream genes, BnaA09g53090D (*BnBDG*) and BnaA04g15380D (*BnFDH*) by qRT-PCR. The results show that the expression levels of *BnBDG* and *BnFDH* were significantly lower in the ZS11-UC1 mutant than in ZS11 (Fig. 4). This result further confirmed that BnaA05g18250D (*BnCFLAP2*) may negatively regulate the expression of *BnBDG* and *BnFDH*, resulting in a defective leaf cuticle, which results in leaf up-curling.

### Agronomic traits

To investigate the effect of the leaf up-curling locus on agronomic traits, 30 flat leaf plants and up-curling leaf plants were randomly from the BC_6_F_2_ population. The results show that for the up-curling leaf plants, plant height, length of main inflorescence, total siliques per plant, 1000-seed weight, and individual plant yield were all significantly lower than the corresponding traits for the flat leaf plants (Table 5). These results indicate that the *BnUC1* locus had negative effects on plant height, and seed weight and yields. There were no significant reductions in stem diameter and branching characters, and no differences in silique extension or seed number per silique in the flat leaf plants compared with the up-curling leaf plants. Therefore, the main agronomic trait of the up-curling leaf mutant was reduced yields.

**Table 5:** Agronomic trait comparisons between plants with the up-curling and flat leaves in the BC_6_F_2_ population

Trait	The flat leaf plant	The up-curling leaf plant
Plant height (cm)	156.16 ± 8.76	132.13 ± 3.28_a_
Branching height (cm)	40.75 ± 4.49	44.31 ± 5.43
Length of main inflorescence (cm)	65.84 ± 2.71	50.38 ± 6.44^a^
Stem diameter (mm)	19.98 ± 3.66	22.35 ± 3.04
Number of first effective branch	7.10 ± 0.94	6.37 ± 0.74
Siliques of main inflorescence	65.30 ± 6.76	59.63 ± 12.88
Total siliques per plant	274.20 ± 85.08	248.88 ± 66.90^a^
Silique length	11.30 ± 0.56	10.45 ± 0.85
Seeds per siliques	30.20 ± 1.88	30.00 ± 3.22
1000-seed weight (g)	4.65 ± 0.12	4.13 ± 0.13^a^
Yield per plant (g)	34.84 ± 10.60	28.20 ± 8.01^a^

^a^indicates significant at the 0.05 probability level by *t*-test. Data are shown as mean ± *SD* (*n* = 30 for each sample)

## Discussion

Leaf is the main photosynthetic organ and its morphology is an important agronomic trait in ideal plant type breeding. Moderate up-curling results in upright leaves with increased light transmittance and light saturation point, which may improve the overall photosynthesis efficiency of a plant population. Appropriate leaf up-curling may help reduce the sun radiation on the leaves, reduce leaf transpiration and water loss in conditions of water shortage, and improve drought tolerance [49]. A rice Rolling-leaf (RL_(t)_) mutant grew slower at the seedling stage than the wild type, but its growth and yield increased significantly during the middle and later growth stages. This example shows the positive effect of leaf curling on plant growth. However, negative effects of leaf curling also have been reported at the individual level [50–52]. Leaf up-curling may reduce the land area occupied by individual plants during their growing period, which may help increase planting density and increase crop unit area yield. Such possible advantages mean it is necessary to further study up-curling leaf development-related genes for plant breeding and to help elucidate this mechanism underlying the leaf trait.

Most studies related to leaf up-curling have focused on monocot crops, and very little is known about this trait in dicot crops. This is the first report on the up-curling locus *BnUC1* in *B. napus.* We found that the *Bnuc1* mutant NIL had an increased net photosynthetic rate at seedling stage, but the total leaf area per plants was reduced, reasonably leading to the lower average individual plant yield compared with the wild type. However, up-curling and upright leaves can change the population light acceptation structure and improve air ventilation, which may help to increase the crop planting density and increase the overall yield per unit area. So, to some extent, the up-curling leaf trait could have a positive effect on breeding and improvement of dicot plants.

The development of genomics and molecular marker technology has led to the use of SNP markers, which have greatly benefitted plant genotyping efforts because of the saturated distinct markers and ideal marker coverage in genomes [53–57]. The genome sequence of *B. napus* (rape) has been published [58]. The *Brassica* 60 K SNP Bead Chip Array has helped advance *B. napus* research efforts, and *Brassica* SNP technology has been used widely in *B. napus* molecular breeding and biology research. In this study, the *BuUC1* locus was mapped to a 2732.549 kb interval on the A05 chromosome by comparing the Brassica 60 K SNP Bead Chip Array genotyping of the 22 up-curling leaf plants with that of the recurrent parent ZS11. The preliminary mapping interval was narrowed using 16 pairs of polymorphic primers for fine mapping. The BC_6_F_2_ population with 654 individuals was used as the isolated population for the fine mapping. Finally, the candidate interval was narrowed to a 54.8 kb region that contained six annotated genes. This strategy to map the target genes is reasonable, low cost, and high efficient.

The molecular mechanism for curling leaf development is complex. It is related to the establishment and maintenance of adaxial-abaxial polarity, in vivo auxin synthesis, development of sclerenchyma, development of cuticle, and other factors, and involves many genes. Among the six genes annotated in the mapping interval in the *B. napus* genome, we identified BnaA05g18250D and BnaA05g18290D as the most probable candidate genes responsible for curling leaf development on the basis of previous findings. BnaA05g18250D is homologous to *CFLAP2* in *A. thaliana*, which can interact with the C-terminal of *CFL1* to regulate the expression of downstream genes *BDG* and *FDH*. BnaA05g18250D also had six protein binding sites that were the same as those in *CFLAP2*, one of which can bind to the C-terminal 119-amino acid region of *CFL1*. Therefore, we inferred that BnaA05g18250D interacts with *CFL1* to affect the development of leaf cuticle, leading to leaf curling. BnaA05g18290D may act as a *RL14* to regulate secondary cell wall formation and water transport to cause the leaf up-curling. The qRT-PCR gene expression analysis, further validated BnaA05g18250D and BnaA05g18290D as candidate genes for *BnUC1*.

## Conclusions

A new leaf mutant with up-curling leaf NJAU5734 was discovered from our *Brassica napus* germplasm. Inheritance studies showed that the up-curling trait was controlled by one dominant locus that we mapped in an interval of 54.8 kb on the *Brassica* A05 chromosome using SNP and SSR markers. Six genes were detected in the mapping interval, and two of them, BnaA05g18250D and BnaA05g18290D, were identified as candidate genes for *BnUC1* based on bioinformatics and gene expression analyses. Thirty individuals were sampled randomly from the BC_6_F_2_ population to investigate the effect of the leaf up-curling locus on agronomic traits. The results indicated that, in general, the up-curling leaf trait had negative effects on agronomic traits. The NIL of the up-curling leaf (ZS11-UC1) was constructed to evaluate the effect of *BnUC1* on photosynthetic efficiency. The results indicated that the up-curling leaf trait locus may be beneficial to improve photosynthetic efficiency and increase planting density.

## Methods

### Plant material and inheritance of the up-curling leaf trait

The double-low oilseed *B. napus* (rape) line NJAU5734 from our germplasm was has an up-curling leaf phenotype. To investigate the genetic control regulation mechanism for this up-curling leaf trait, we crossed NJAU5734 with the canola variety Zhongshuang 11 (ZS11) to produce an F_1_ population. The F_1_ individuals were selfed to generate F_2_ mapping populations and backcrossed with ZS11. The selfing and backcrossing populations were observed for segregation ratio of plants with normal flat leaf to plants with up-curling leaf. The sixth backcross generation (BC_6_) and BC_6_F_2_ family populations were used for preliminary mapping and fine mapping of the up-curling leaf locus. All materials were grown at the same density in fields of the Jiangpu Experimental Station at the Nanjing Agricultural University (Jiangsu Province, China). The plants were sown uniformly in rows 2.5 m in length with 15 individuals in each row and 0.4 m spacing between rows. Chi-square tests were performed on the segregation data to determine the genetic regulation for the up-curling leaf trait.

### Single nucleotide polymorphism (SNP) analysis

The BC_6_ family population was identified using SNP markers. Twenty-two BC_6_ up-curling leaf plants and the two parents were genotyped using a Brassica 60 K SNP Bead Chip Array (Illumina, Inc), to detect the differential segments in the backcross family population from ZS11. This information also was the basis for building NILs for ZS11. The SNP markers were named using “M” plus the index numbers assigned by Genome Studio v2011.1 (Illumina, Inc.). The SNP analysis was the same as that in a previous study [59].

The obtained SNPs of the 22 up-curling leaf plants were compared with the SNPs of the recurrent parent ZS11 on 19 chromosomes to find the locus controlling the up-curling leaf trait. We found a BC_6_ plant (number ZS11-UC-6) that had only one differential chromosome segment, located in an interval of 2732.549 kb between SNP markers M10649 and M10888 on the A05 chromosome of *B. napus*, and identified this segment as harboring the up-curling leaf locus.

### Mapping the up-curling leaf locus

The individual ZS11-UC-6 plant was selfed, generating a BC_6_F_2_ family population with 654 individuals that were used to fine map the up-curling leaf locus using simple sequence repeat (SSR) markers.

First, we downloaded the *B. napus* genomic sequence between SNP markers M10888 and M10649 from the *Brassica napus* Genome Browser (http://www.genoscope.cns.fr/brassicanapus/cgi-bin/gbrowse/colza/) and the *Brassica* database (BRAD; http://brassicadb.org/brad/). Then, SSR loci were detected using SSR Hunter 1.3 [60], and SSR marker primers were designed using Primer Premier 5.0 [61]. A total of 201 primer pairs were designed to map the up-curling leaf locus. The mapping interval for the up-curling leaf locus was gradually reduced using the mapping results of the BC_6_F_2_ population. Finally, a fine linkage map for the up-curling locus was constructed using JoinMap 4.1 with the polymorphic SSR markers [62] that were detected by screening the ZS11-UC-6 and parent (ZS11 and NJAU5734) genomic segments.

Sixteen polymorphic SSR makers were found among those detected using the 201 primer pairs (Additional file 5: Table S2). These markers were developed gradually as the region of the sequence that contained the dominant up-curling leaf locus narrowed. The polymerase chain reaction (PCR) conditions for the molecular marker experiments were as follows: denaturation at 94 °C for 5 min, followed by 35 cycles of 94 °C for 30 s, annealing for 30 s (at the annealing temperature of each SSR marker), and 72 °C for 30 s, and a final extension step at 72 °C for 10 min. The total DNA extraction and linkage map construction were performed as described previously [63].

### Genes in the mapping interval

Chromosome sequences of two *Brassica* genomes (*B. rapa* and *B. napus* cv*.* ‘Darmor*-bzh*’) homologous to the fine mapping interval on the A05 chromosome were downloaded from BRAD (http://brassicadb.org/brad/) and http://www.genoscope.cns.fr/brassicanapus/ to identify genes in the mapping interval [64, 65]. The homologous sequences were aligned using BLASTN (http://blast.ncbi.nlm.nih.gov/) and their similarity was assessed by dot matrix analysis to help understand the mapping interval in terms of genome evolution. The genes detected in the mapping interval were annotated according to the annotations of the *B. napus* cv*.* ‘Darmor-*bzh*’ genes.

### Near-isogenic line of the up-curling leaf plant

The effects of the up-curling leaf locus on phenotypic traits were evaluated by quantitative real-time PCR (qRT-PCR), measuring chlorophyll content, and determining leaf photosynthetic characteristics of the NIL of the up-curling leaf (ZS11-UC1), which was obtained from the BC_6_F_2_ family population. ZS11-UC1 has a 438.99-kb segment that is different from the corresponding segment of ZS11.

### Quantitative RT-PCR analysis

The expression levels of genes in the fine mapping interval were compared between the two NILs ZS11 and ZS11-UC1. Leaves from four plants at the five-leaf stage in each of the NILs were sampled for qRT-PCR. Total RNA was extracted using TRIzol reagent (Sigma; http://www.sigmaaldrich.com/). After digestion with DNase I, 1 μg RNA was reverse-transcribed into cDNA using a Reverse Transcription System (Takara, Tokyo, Japan). The cDNA was used as the template for qRT-PCR analysis with specific primers (Additional file 6: Table S3). We aligned the gene sequences with the annotated homologous BODYGUARD (*BDG*) and FIDDLEHEAD (*FDH*) in *Brassica napus* Genome Brower using BLAST, and found that the BnaA09g53090D gene was most similar to *AtBDG* (AT1G64670) and the BnaA04g15380D gene was most similar to *AtFDH* (AT2G26250). Then, we designed specific primers to the corresponding genes toanalyze their expression. The expression of each gene in the different RNA samples was normalized to the expression of actin, which was used as a housekeeping gene (Additional file 6: Table S3). The qRT-PCRs were carried out with SYBR Green Real-time PCR Master mix using a CFX96–2 PCR machine (BIO-RAD, USA) and the relative gene expression levels were analyzed as described previously [66]. Four biological replicates were used. Relative expression levels were calculated using the 2^−ΔΔCt^ method with actin as an internal control.

### Determination of chlorophyll content and photosynthetic efficiency

Chlorophyll (Chl) was extracted from 0.2 g of fresh leaves with 50 ml of 80% acetone for content determination using an Alpha-1500 spectrophotometer (LASPEC, Shanghai, China). Chl a and Chl b content was measured as described previously [67, 68]. Each experiment had 15 biological replicates.

The photosynthetic characteristics of the NILs were determined using a Li-Cor 6400 portable photosynthesis system (Li-Cor Inc., Lincoln, NE, USA) with the built-in light source set at 1000 μmol photons m^− 2^ s^− 1^, for net photosynthetic efficiency and stomatal conductance. All measurements were done between 09:00 am and 11:00 am, with the leaf temperature adjusted to 23 °C [68]. Each experiment had six biological replicates.

### Agronomic traits

To evaluate agronomic efficiency of the up-curling leaf plants and the flat leaf plants, 30 individuals were sampled randomly from the BC_6_F_2_ population*.* The traits investigated were as follows: plant height, branching height, length of main inflorescence, stem diameter, number of first effective branches, siliques of main inflorescence, total siliques per plant, silique length, seeds per siliques, 1000-seed weight, and yield per plant. The mean values of all the agronomic traits were compared between the up-curling leaf and the flat leaf plants by *t*-tests.

## Additional files

Additional file 1: **Figure S1.** Leaf morphology of the near-isogenic line (ZS11-UC1). (DOCX 50 kb)

Additional file 2: **Figure S2.** The melting curves of 8 genes and the housekeep gene Actin. (DOCX 99 kb)

Additional file 3: **Figure S3.** The standard curves for the amplification of 8 genes and the housekeep gene Actin. (DOCX 54 kb)

Additional file 4: **Table S1.** The amplification efficiency of each primers couple. (DOCX 14 kb)

Additional file 5: **Table S2.** The designed SSR markers used in this study. (DOCX 30 kb)

Additional file 6: **Table S3.** The designed qRT-PCR primers used in this study. (DOCX 14 kb)

## Data Availability

All the data generated or analyzed during this study are included in this published article and its supplementary information files.

## Abbreviations

AGAL3: α-Galactosidase
*B. napus*: *Brassica napus*;
*BDG*: BODYGUARD
*BnUC1*: Up-curling leaf locus in *B. napus* genome
*Bnuc1*: Up-curling leaf mutant
*CFLAP2*: AtCFL1 associated protein
Chl: Chlorophyll
*FDH*: FIDDLEHEAD
HD-ZIP III: Class III HOMEODOMAIN LEUCINE-ZIPPER
NIL: Near Isogenic line
PCR: Polymerase chain reaction
qRT-PCR: Quantitative real-time PCR
*RL14*: Rolling-leaf 14
SNP: Single nucleotide polymorphism
SPRY: SPla and RYanodine receptor
SSR: Simple sequence repeat
TAIR: The Arabidopsis Information Resource
ZS11: Zhongshuang 11
ZS11-UC1: Near isogenic line with the up-curling leaf
