# Supplementary figures and images for: Fine mapping of an up-curling leaf locus (BnUC1) in Brassica napus
Source: BMC Plant Biol. 2019 Jul 19;19:324. doi: 10.1186/s12870-019-1938-0 (PMC6642557; doi:10.1186/s12870-019-1938-0)

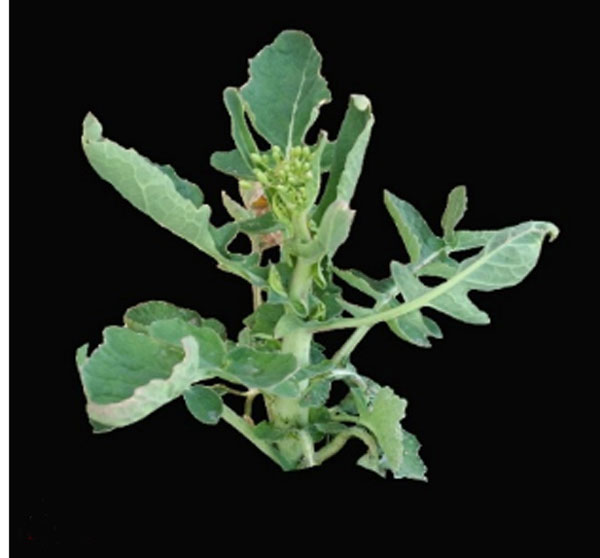


**Additional file 1: Figure S1** Leaf morphology of the near-isogenic line (ZS11-UC1).

Supplement: Supplementary file 1 — Figure S1. Leaf morphology of the near-isogenic line (ZS11-UC1). (DOCX 50 kb) [file 12870_2019_1938_MOESM1_ESM.docx]
