# Supplementary material for: Fine mapping of an up-curling leaf locus (BnUC1) in Brassica napus
Source: BMC Plant Biol. 2019 Jul 19;19:324. doi: 10.1186/s12870-019-1938-0 (PMC6642557; doi:10.1186/s12870-019-1938-0)
Supplement: Supplementary file 2 — Figure S2. The melting curves of 8 genes and the housekeep gene Actin. (DOCX 99 kb) [file 12870_2019_1938_MOESM2_ESM.docx]

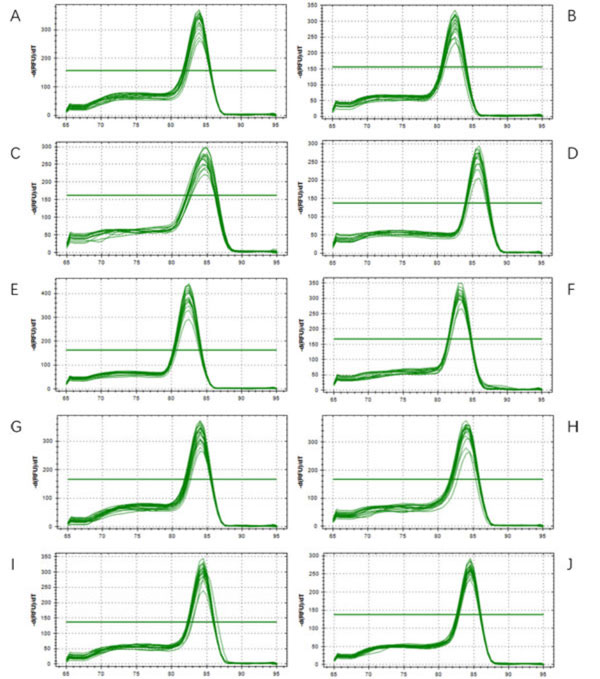


**Additional file 2: Figure S2** The melting curves of 8 genes and the housekeep gene Actin. A and G denote the melting curves of the housekeep gene *BnActin* in different plates; B-F denote the melting curves of BnaA05g18240D, BnaA05g18250D, BnaA05g18260D, BnaA05g18270D and BnaA05g18280D, respectively; H-J denote the melting curves of BnaA05g18290D, *BnBDG* and *BnFDH*, respectively.
