# Supplementary material for: Fine mapping of an up-curling leaf locus (BnUC1) in Brassica napus
Source: BMC Plant Biol. 2019 Jul 19;19:324. doi: 10.1186/s12870-019-1938-0 (PMC6642557; doi:10.1186/s12870-019-1938-0)
Supplement: Supplementary file 3 — Figure S3. The standard curves for the amplification of 8 genes and the housekeep gene Actin. (DOCX 54 kb) [file 12870_2019_1938_MOESM3_ESM.docx]

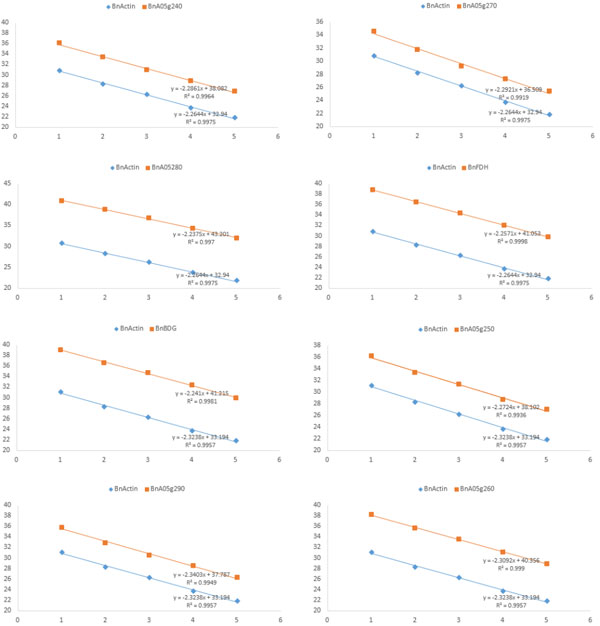


**Additional file 3: Figure S3** The standard curves for the amplification of 8 genes and the housekeep gene Actin. The gradient of the reverse-transcribed cDNA was diluted 5,5^2^,5^3^,5^4^,5^5^.
