# Supplementary material for: Fine mapping of an up-curling leaf locus (BnUC1) in Brassica napus
Source: BMC Plant Biol. 2019 Jul 19;19:324. doi: 10.1186/s12870-019-1938-0 (PMC6642557; doi:10.1186/s12870-019-1938-0)
Supplement: Supplementary file 4 — Table S1. The amplification efficiency of each primers couple. (DOCX 14 kb) [file 12870_2019_1938_MOESM4_ESM.docx]

**Additional file 4: Table S1** The amplification efficiency of each primers couple.

| Name of Genes | Equation | R^2^ | Amplification efficiency (%) |
| --- | --- | --- | --- |
| BnaA05g18240D | Y=-2.2861X+38.082 | 0.9964 | 102.18% |
| BnaA05g18250D | Y=-2.2724X+38.102 | 0.9936 | 98.91% |
| BnaA05g18260D | Y=-2.3092X+40.356 | 0.9990 | 100.77% |
| BnaA05g18270D | Y=-2.2921X+36.509 | 0.9919 | 101.81% |
| BnaA05g18280D | Y=-2.2375X+43.201 | 0.9970 | 104.99% |
| BnaA05g18290D | Y=-2.3403X+37.787 | 0.9949 | 103.04% |
| BnBDG | Y=-2.2410X+41.215 | 0.9981 | 104.99% |
| BnFDH | Y=-2.2571X+41.053 | 0.9988 | 104.02% |
| BnActin | Y=-2.3238X+33.194  Y=-2.2644X+32.940 | 0.9957  0.9975 | 99.89%  103.55% |

The gradient of the reverse-transcribed cDNA was diluted 5,5^2^,5^3^,5^4^,5^5^.
