# Supplementary material for: Fine mapping of an up-curling leaf locus (BnUC1) in Brassica napus
Source: BMC Plant Biol. 2019 Jul 19;19:324. doi: 10.1186/s12870-019-1938-0 (PMC6642557; doi:10.1186/s12870-019-1938-0)
Supplement: Supplementary file 5 — Table S2. The designed SSR markers used in this study. (DOCX 30 kb) [file 12870_2019_1938_MOESM5_ESM.docx]

**Additional file 5: Table S2** The designed SSR markers used in this study.

| Name of Primers | Sequence of primers | Product length | Chromosome  Location |
| --- | --- | --- | --- |
| BnA05A3-F | CCGAGCAGCCAGCAAATA | 206 | 11205649 |
| BnA05A3-R | AACGAGCAGATCAATCAATAC |  |  |
| BnA05A89-F | TTGAGGCTTTCCGTGGGA | 247 | 11232196 |
| BnA05A89-R | AGGTTCGGTCAGTGGTGC |  |  |
| BnA05A195-F | GAGATGGAACTTCACTTG | 195 | 11258492 |
| BnA05A195-R | GCTTTAATCAGCCACTAC |  |  |
| BnA05A244-F | AGAATACAAACGATCACG | 199 | 11265977 |
| BnA05A244-R | AAGAGGAAATAAAAGCAG |  |  |
| BnA05A295-F | CTGTACCGTTATCTAGCATT | 194 | 11274392 |
| BnA05A295-R | AAGTGAAGCGTACCTTTG |  |  |
| BnA05A336-F | AGCAGGCTAACAGGGAGG | 180 | 11285958 |
| BnA05A336-R | TGCGCTGAGTGGAGTAAAA |  |  |
| BnA05A432-F | TTTACCAAAATGAGAAATGTAC | 190 | 11321609 |
| BnA05A432-R | AACGAACCCGACACTTTA |  |  |
| BnA05A506-F | TTGTCCATTCTTGATTTCCCTT | 277 | 11339735 |
| BnA05A506-R | CCGCTCCCAAACACCATT |  |  |
| BnA05A576-F | AAGTTAGGCGTTGGATTA | 208 | 11353941 |
| BnA05A576-R | CACAATGTCTACGGTTCT |  |  |
| BnA05A628-F | CAGATCGGAAGGGCAAAG | 186 | 11362089 |
| BnA05A628-R | CCTCACAAACCACCAGCA |  |  |
| BnA05A703-F | TGGCACAGAACTGTAAAT | 217 | 11377068 |
| BnA05A703-R | GTGAAAAGAAACTTGGTAAT |  |  |
| BnA05A787-F | AGACAACATAAAGTTCCAGC | 214 | 11394699 |
| BnA05A787-R | TACGCAAGGAATGAAAGA |  |  |
| BnA05A876-F | TGATCTACGGTTTCATCCC | 223 | 11411234 |
| BnA05A876-R | ATGCGTTATTGAAGAGGATT |  |  |
| BnA05A934-F | GACCTGACCCTAGTTCCA | 187 | 11424025 |
| BnA05A934-R | CCTCTTGCTAAGATCCCT |  |  |
| BnA05A996-F | TTTTAGTGCTTGTTTTCT | 204 | 11437945 |
| BnA05A996-R | TTTGGAGTAACACTATTTCT |  |  |
| BnA05A1055-F | CACAAATCAAACTTCATCTGCCT | 190 | 11449561 |
| BnA05A1055-R | TTGGACGCCCTAACTCGC |  |  |
| BnA05A1118-F | AATCAAAGCAAATCACAA | 196 | 11469701 |
| BnA05A1118-R | CTCTTCCTTTTATGTAATACTT |  |  |
| BnA05A1172-F | ATCATAAAAGCGGGCATA | 206 | 11479463 |
| BnA05A1172-R | GTCAGAATGGGGACCTTA |  |  |
| BnA05A1235-F | CCAAATGAAGCCAAACTAT | 243 | 11494750 |
| BnA05A1235-R | ACTTGACCATCACCCTCC |  |  |
| BnA05A1284-F | TCCTACGGTGGCTATTTG | 199 | 11508012 |
| BnA05A1284-R | TCACTTGAGGGACACTTCT |  |  |
| BnA05A1351-F | ATTATGGGTCTAGGAAAT | 220 | 11524657 |
| BnA05A1351-R | AATGATGTCTATAGTGGC |  |  |
| BnA05A1406-F | GGAAAATGATGAAAAGTC | 214 | 11538329 |
| BnA05A1406-R | AGAATGAGTACATGCAGAG |  |  |
| BnA05A1482-F | TCCATAACGAGCAGAGCA | 226 | 11553654 |
| BnA05A1482-R | CCATTTCTTGAGTCCCAGT |  |  |
| BnA05A1504-F | AAGAAGTGACTAAGTCCATAA | 202 | 11557118 |
| BnA05A1504-R | TTCGTCCAACTCATAACA |  |  |
| BnA05A1581-F | CCATTTTCTTCACTGAAA | 206 | 11572806 |
| BnA05A1581-R | ATTGGTGATAATAGATGTGA |  |  |
| BnA05A1622-F | TGTTTACCGATTCCAAGA | 181 | 11583856 |
| BnA05A1622-R | GAGCACGAGAAAGCGAGT |  |  |
| BnA05A1701-F | GCCGATAAAGATGATAGA | 195 | 11601440 |
| BnA05A1701-R | GATTCGAGAAGTTGTAGATG |  |  |
| BnA05A1782-F | TTACGCAGTACGATACAA | 185 | 11618059 |
| BnA05A1782-R | TCATAACTCGCAGCTCTA |  |  |
| BnA05A1827-F | TGCGAAGACAACAAATAA | 191 | 11628433 |
| BnA05A1827-R | CTTGAGACAAAACCGAAT |  |  |
| BnA05A1890-F | CATTTGCGTAATTTAGGT | 205 | 11638695 |
| BnA05A1890-R | ATTGGAATCAGACACTTT |  |  |
| BnA05A1949-F | TCGTGACAAAATTAAGCG | 204 | 11649563 |
| BnA05A1949-R | AAAGGACTGGAAATGGAT |  |  |
| BnA05A2001-F | AATGCCATCTGTTTTACG | 186 | 11658947 |
| BnA05A2001-R | TATCCAACAAATTCCTCTTT |  |  |
| BnA05A2044-F | CTTTGGAACATACATCT | 198 | 11668477 |
| BnA05A2044-R | AAATACTTATAGGCATCA |  |  |
| BnA05A2118-F | TTGCTACAAAGAAGAATA | 205 | 11694476 |
| BnA05A2118-R | TAATCAAAGGTGTAAACA |  |  |
| BnA05A2217-F | AGTTATTTCAGTTTATGG | 199 | 11714581 |
| BnA05A2217-R | TTAGTGAGAATCCTTACA |  |  |
| BnA05A2320-F | GATACATGGCTCCAGAGG | 203 | 11739938 |
| BnA05A2320-R | GGATAAGGCATAGCACAAC |  |  |
| BnA05A2398-F | CCCAGACGAGCAGTGAGA | 218 | 11756253 |
| BnA05A2398-R | AGGCTTGCTGGTTTACGC |  |  |
| BnA05A2488-F | ACGGTTAGGAAAGGGAGT | 207 | 11774279 |
| BnA05A2488-R | CCGATAGGTGGTCAGTGTAT |  |  |
| BnA05A2594-F | GAGCGGTCAAATAATCAA | 230 | 11803988 |
| BnA05A2594-R | AACGACCAAACACCAAAG |  |  |
| BnA05A2666-F | TGCGTTATTGAGCAGTGAG | 185 | 11828775 |
| BnA05A2666-R | GTGTCGAGGATGGAGGTG |  |  |
| BnA05A2758-F | TCTCGTGGTCTCATAGTT | 183 | 11850812 |
| BnA05A2758-R | TATACTAGGTGTTTTGCC |  |  |
| BnA05A2866-F | ATGTGCGTAAGAACCATAA | 241 | 11870094 |
| BnA05A2866-R | GCAAGACGGTCCTGTAGTC |  |  |
| BnA05A2959-F | GCGGTTGTTGGAGTTGGT | 231 | 11892583 |
| BnA05A2959-R | ATGGGAAGCAGATGTACGAG |  |  |
| BnA05A3063-F | TTCTGTTTTGCATTACAA | 209 | 11912538 |
| BnA05A3063-R | AGAGCATCAAGATCATTT |  |  |
| BnA05A3158-F | TGGATGTTTGAAGATTTT | 194 | 11931718 |
| BnA05A3158-R | CCATAGGACCTAAGGACT |  |  |
| BnA05A3263-F | AAGCCACCATCTCCTTCT | 197 | 11949603 |
| BnA05A3263-R | TTCATCATTCTTCCTCGA |  |  |
| BnA05A3364-F | CCTTCAATAAAGAAAATG | 204 | 11967294 |
| BnA05A3364-R | ATAACCATCAATGAGAAA |  |  |
| BnA05A3475-F | CTGAATACCTTCCAGCAC | 212 | 11989888 |
| BnA05A3475-R | CCACAATCTGTAATCCAAT |  |  |
| BnA05A3586-F | CATTCCACCTCCTCCTTTCA | 198 | 12012845 |
| BnA05A3586-R | CAGATCGCAATATCTCCTTCC |  |  |
| BnA05A3689-F | TGTTTCTTATTCCTCCCTC | 209 | 12030998 |
| BnA05A3689-R | GTTAGTCCCATCTGTCCC |  |  |
| BnA05A3799-F | ACAAGTGGTTCCTGCTCC | 183 | 12051506 |
| BnA05A3799-R | CCAACCTAAGTCCCGTAAT |  |  |
| BnA05A3893-F | GACATGCCAATTACCACC | 229 | 12127334 |
| BnA05A3893-R | AACGACAGATGCGTTTCA |  |  |
| BnA05A3989-F | GCCGTCGCTAGTTTAGGC | 180 | 12151500 |
| BnA05A3989-R | TCGGTGGAAATGGAATGA |  |  |
| BnA05A4100-F | TTGGGAAGAAACAGCATA | 210 | 12176594 |
| BnA05A4100-R | GTGTAACTTTTGTGGGAT |  |  |
| BnA05A4209-F | ATAATTTGTGGAGGAGATGCG | 226 | 12201112 |
| BnA05A4209-R | TGGCTTGGAACCTGACCC |  |  |
| BnA05A4308-F | AGAAGCCTTTCCTTACTA | 221 | 12222859 |
| BnA05A4308-R | ACTAAAATAAACGAAGCA |  |  |
| BnA05A4412-F | CGCGGGGTTGTTGGATTA | 180 | 12258992 |
| BnA05A4412-R | TGGGTCGGTTCGGTTTGT |  |  |
| BnA05A4577-F | AGAAAAGAAAAGTTTGCAC | 186 | 12330875 |
| BnA05A4577-R | CAGCACGACCCATAACAC |  |  |
| BnA05A4741-F | AAACTTGTTGCTTTTACC | 195 | 12372153 |
| BnA05A4741-R | CACCTGCTCACTCCTCTA |  |  |
| BnA05A50-F | AAAGCACAGATAAGTAATACAAG | 185 | 12383360 |
| BnA05A50-R | AAGTTATCCCAACACCAG |  |  |
| BnA05A264-F | CTTGCGGCTATGCTCTGC | 186 | 12452275 |
| BnA05A264-R | GGCCTCCACCTCAACGAA |  |  |
| BnA05A326-F | CCACAAACCTATCTGAACCCTG | 249 | 12464511 |
| BnA05A326-R | GCCCAAGTCCAAGCCAAA |  |  |
| BnA05A451-F | TGGCAATCTTCTGCTCTT | 192 | 12488916 |
| BnA05A451-R | ATTTCCACTCGACCGTTT |  |  |
| BnA05A681-F | CACATTTTCCCTAACTCG | 183 | 12547731 |
| BnA05A681-R | TCTGCCACCTTGTCTCAC |  |  |
| BnA05A821-F | ACATGAGTCTGTGGCGATAA | 198 | 12583103 |
| BnA05A821-R | CGTGAAATCAAAGTAGGTGC |  |  |
| BnA05A1001-F | ACATGAGTCTGTGGCGATAA | 198 | 12596333 |
| BnA05A1001-R | CGTGAAATCAAAGTAGGTGC |  |  |
| BnA05A1091-F | CAAGAAAGCCTTAGACAA | 261 | 12606429 |
| BnA05A1091-R | AGAGCCGAGGTCACATAA |  |  |
| BnA05A1165-F | CCCTCTACTGACTTTATTTCT | 206 | 12634849 |
| BnA05A1165-R | CACTATTCGCCTACACTTT |  |  |
| BnA05A1268-F | TTGTGGGACAATGGTGAA | 225 | 12660391 |
| BnA05A1268-R | ATTGGTGAGCAAGGCAGT |  |  |
| BnA05A1396-F | TTGTATGTATTCTTAGGATTAG | 277 | 12699061 |
| BnA05A1396-R | TAACAAACAAAGATGACG |  |  |
| BnA05A1618-F | CTATTTTCCTACTGGCTCT | 185 | 12765305 |
| BnA05A1618-R | TTGATTCCAATTCTGCTA |  |  |
| BnA05A1727-F | TTTCGAGAATTAGTTGCC | 205 | 12788847 |
| BnA05A1727-R | GTCACTTTAGCGAGGTTT |  |  |
| BnA05A1821-F | TCGTTGAAACTGCGACTG | 233 | 12809850 |
| BnA05A1821-R | TGGAGGCACAATTCTATC |  |  |
| BnA05A2125-F | GCACCGAAGAAGACGACA | 176 | 12872802 |
| BnA05A2125-R | TCTGAGCCGACCGCAACT |  |  |
| BnA05A2296-F | AATGGATTAAACGAGGGT | 258 | 13002168 |
| BnA05A2296-R | GAGTTCTTCATCGGTTGG |  |  |
| BnA05A2331-F | CTTCAAATACAGACAAATTAT | 217 | 13010175 |
| BnA05A2331-R | GAACCCACTCCAAAACCG |  |  |
| BnA05A2468-F | GTGATGTCCCTCCTTCTGGTG | 194 | 13036929 |
| BnA05A2468-R | CTCCTCGTCGTCTTCTTCTCC |  |  |
| BnA05A2532-F | AAATCAAATACACCCTAAC | 182 | 13108969 |
| BnA05A2532-R | ATGGAAATGTAGAGGAGA |  |  |
| BnA05A2541-F | TTTTGTCATCCTTGTAAC | 239 | 13111766 |
| BnA05A2541-R | CTACTAGCCTTGACTTTT |  |  |
| BnA05A2651-F | ATAAATCAACTGGATATGG | 184 | 13140570 |
| BnA05A2651-R | GCTGTCTGACTTGGAAAA |  |  |
| BnA05A2765-F | GCTTTGTAAACCGCCACT | 205 | 13167067 |
| BnA05A2765-R | TTCATCAATCCGCCATCT |  |  |
| BnA05A2794-F | AGGTGAGTCAGTGTTCCATC | 223 | 13178006 |
| BnA05A2794-R | CCAAGGGTTTCAGTTAGA |  |  |
| BnA05A2870-F | GGTGAGACGCGATGAAGA | 262 | 13197479 |
| BnA05A2870-R | GCAAAAGGGGAAAAGTAAA |  |  |
| BnA05A2942-F | ATGAGGCAACATTAAGCG | 213 | 13211912 |
| BnA05A2942-R | AAACCAACAAGGAATAGGA |  |  |
| BnA05A2964-F | TTGAGGGTACTTCCATACA | 238 | 13216697 |
| BnA05A2964-R | CAAAACACGAAACCAGAG |  |  |
| BnA05A2987-F | AGCCTTAATGGTTGTAGC | 192 | 13222221 |
| BnA05A2987-R | ATGGGATCAGATTCTTCA |  |  |
| BnA05A3063-F | CCACTTCCGCAACTCGTA | 177 | 13250253 |
| BnA05A3063-R | TTTTCCGGCCATCCTATT |  |  |
| BnA05A3109-F | CTATAATCAAACAAGCCACG | 242 | 13260998 |
| BnA05A3109-R | CTGAGATGTCGGAGGAGG |  |  |
| BnA05A3120-F | TCAACGAGGAGACTCTACAGCA | 234 | 13262892 |
| BnA05A3120-R | CACGCAACACCTTCTTACGATA |  |  |
| BnA05A3251-F | CCTACATAGGATGCTGACTG | 216 | 13314222 |
| BnA05A3251-R | GGAGGTTTATTTTACCCACA |  |  |
| BnA05A3267-F | AGTTGCAGGTTTGTGAGT | 180 | 13317553 |
| BnA05A3267-R | AAGCCTAAGGGTTTGTTA |  |  |
| BnA05A3404-F | GAATATGCCATTTCCAGA | 232 | 13351593 |
| BnA05A3404-R | GACCCTAGCTTTAGTGTTTT |  |  |
| BnA05A3444-F | AAATGAATCTGACAAAACCCTC | 290 | 13362092 |
| BnA05A3444-R | TCCCCTAACCCGTGAAAA |  |  |
| BnA05A3445-F | AAACATAGGAGGTGAGGGAA | 275 | 13362241 |
| BnA05A3445-R | GTCATCCGAAGGAGGGTC |  |  |
| BnA05A3446-F | GCTAGGGTTTCTCCTTCTCG | 206 | 13362315 |
| BnA05A3446-R | ACAACGGCTTTGTCATCC |  |  |
| BnA05A3573-F | TCAACGAACTCAGTCCAC | 236 | 13393702 |
| BnA05A3573-R | TCTCCACAGCAAAGGTAA |  |  |
| BnA05A3629-F | CATGACGAAGCTGCTAAC | 202 | 13411008 |
| BnA05A3629-R | ATGCCAATCAATCAACAA |  |  |
| BnA05A3684-F | AGAAGGAGACTCTGTGATT | 175 | 13419206 |
| BnA05A3684-R | ACGGAAAGACCTTATGTA |  |  |
| BnA05A3691-F | TAAACCCAAGACTTCACC | 182 | 13420052 |
| BnA05A3691-R | AATGGAGTTCCAATCACA |  |  |
| BnA05A3692-F | AGCCAAGACTTCACCAAA | 297 | 13420065 |
| BnA05A3692-R | GCTAATCCCTCTAACCTATT |  |  |
| BnA05A3693-F | AGTGTTTAGAGGCAGGAG | 253 | 13420099 |
| BnA05A3693-R | ACCACAACTTAGCAATCT |  |  |
| BnA05A3694-F | ATAGACTTGCTCATTTAGG | 240 | 13420883 |
| BnA05A3694-R | TACAAACGATGTGGAGAT |  |  |
| BnA05A3736-F | TAGTTGAAATGATAAAAGTC | 229 | 13429927 |
| BnA05A3736-R | GTAGAAAAGGAAACCGTA |  |  |
| BnA05A3767-F | GGAAAAGGAATCATACTCA | 264 | 13434168 |
| BnA05A3767-R | AGTCTTCTTGCCAATAAAC |  |  |
| BnA05A3825-F | TTAGGATTTTAGAAAAGG | 192 | 13447000 |
| BnA05A3825-R | ATTGTGACATGTGGTAGA |  |  |
| BnA05A3846-F | ATAAGATGGTTTTCTGAG | 210 | 13449177 |
| BnA05A3846-R | CATATTTGCACTGAGTAG |  |  |
| BnA05A3865-F | TTACGGATAAAATCATCTC | 224 | 13452854 |
| BnA05A3865-R | TACTCCCCAAAATAGCAT |  |  |
| BnA05A3882-F | TTCGACCTTCTCGTACTTC | 186 | 13468626 |
| BnA05A3882-R | CTGAGCCATTGGACAACT |  |  |
| BnA05A3891-F | TTTTGGGAAGAAGGGATG | 215 | 13480451 |
| BnA05A3891-R | CACCAGGAACACTCAAGAAC |  |  |
| BnA05A3912-F | TGTTACAATACGAGGTCTTCCG | 197 | 13486058 |
| BnA05A3912-R | GGCTATGCTTGATGTGAGGG |  |  |
| BnA05A3922-F | GAGTAACAATTAAGCGAA | 247 | 13487083 |
| BnA05A3922-R | AAACTGTCAAATCCATAG |  |  |
| BnA05A3934-F | GATTCTACCCTTAGTTTT | 216 | 13488905 |
| BnA05A3934-R | GTCGTCTGATAATCTTTT |  |  |
| BnA05A3938-F | GTGGTTTGGGTTTGGTAT | 211 | 13489541 |
| BnA05A3938-R | AACGGTTGAGCTTAGTAGGT |  |  |
| BnA05A3943-F | TAAAACTCAGAATCCGAC | 208 | 13490286 |
| BnA05A3943-R | CTAATCCAAATCAAACAA |  |  |
| BnA05A3949-F | TCATAGTTTTCAGGTTTC | 205 | 13490954 |
| BnA05A3949-R | TTCCGTAACATACATTTC |  |  |
| BnA05A3966-F | TGGAGGGGCGGACACTAT | 201 | 13493777 |
| BnA05A3966-R | CCCGATGAAAATAACTATGAAG |  |  |
| BnA05A3981-F | AGAATAAGCAACAAATGG | 224 | 13498758 |
| BnA05A3981-R | CAAATCATCCTCGTCAAT |  |  |
| BnA05A3982-F | CTCCCTTCAACTTTATTCCT | 228 | 13499540 |
| BnA05A3982-R | TGTCTCAGCAGCATCTCC |  |  |
| BnA05A4001-F | GCACAGGTGTATGATAAT | 180 | 13503588 |
| BnA05A4001-R | CTAGATAAACAGAGTCGTC |  |  |
| BnA05A4008-F | GAGACGCTGCTTTGAACA | 205 | 13505012 |
| BnA05A4008-R | TCCCATTAGATTCCACCT |  |  |
| BnA05A4014-F | ACAAAATCCATTAGAAAC | 189 | 13506328 |
| BnA05A4014-R | AAGTAATGTTGGAGTATCA |  |  |
| BnA05A4017-F | CCATAAGCTCCGATAGTGA | 196 | 13507382 |
| BnA05A4017-R | TTGCGTCTGAGTTAGTCCA |  |  |
| BnA05A4030-F | GTGAATGCCATAACCAAA | 205 | 13510607 |
| BnA05A4030-R | GTTGTAAACTAATGGGAGAA |  |  |
| BnA05A4041-F | GATACACGGCGGTTGATG | 245 | 13513897 |
| BnA05A4041-R | AAAGCGAAAGAAAGGTCAA |  |  |
| BnA05A4056-F | TCTTGCCTTTTCCTCTTC | 244 | 13517412 |
| BnA05A4056-R | GTTCCTTGATTCCACCAG |  |  |
| BnA05A4072-F | AGCACCACAACTGAACTG | 192 | 13523815 |
| BnA05A4072-R | AAACTCAACTTTGCCTTC |  |  |
| BnA05A4073-F | AGCACCACAACTGAACTG | 192 | 13523826 |
| BnA05A4073-R | AAACTCAACTTTGCCTTC |  |  |
| BnA05A4074-F | TTACCTCGATGATGTTCTC | 257 | 13523871 |
| BnA05A4074-R | ACTTTGCTGCTACTTTCTT |  |  |
| BnA05A4090-F | GAGAACTTACAAGAACCCTA | 192 | 13527660 |
| BnA05A4090-R | GATTCAGTGAATAAACCC |  |  |
| BnA05A4099-F | GGAAATGAAAATGGAACA | 277 | 13529007 |
| BnA05A4099-R | TAGAATGACCTTTTGACG |  |  |
| BnA05A4105-F | CCAAAGGGAGCAGGAAAG | 209 | 13530838 |
| BnA05A4105-R | CGAAGGAGGAATGGGAAC |  |  |
| BnA05A4111-F | CCCGAGAAAGGAAACTCT | 257 | 13532806 |
| BnA05A4111-R | GAGGCAGTCACTAATGGAAT |  |  |
| BnA05A4123-F | CAACCACCCTGGCACAAC | 214 | 13537117 |
| BnA05A4123-R | GCTCCGCAAGGACATTCA |  |  |
| BnA05A4127-F | AAGGAGGAATGGGAACGA | 247 | 13538848 |
| BnA05A4127-R | AGCCGACTCACCTTGGAC |  |  |
| BnA05A4137-F | AACAGTTCGTTGTGGTGGTA | 247 | 13542532 |
| BnA05A4137-R | CAAATATCCGGCAAGTCC |  |  |
| BnA05A4142-F | TAACGCTGTAATTTTATAATGG | 244 | 13543060 |
| BnA05A4142-R | CTCGTATGGGAGGTGGAC |  |  |
| BnA05A4160-F | TGATGGTGCCTCCGAGTG | 284 | 13546466 |
| BnA05A4160-R | TTCCAGTTCCGATGTGCC |  |  |
| BnA05A4164-F | TGCTCTGCCTCCCGTAAC | 265 | 13547111 |
| BnA05A4164-R | TTGCTCGGACTTGCCATC |  |  |
| BnA05A4177-F | ATTGGAATAACATAAGAG | 207 | 13549262 |
| BnA05A4177-R | TGATTATAGTATGCCTTC |  |  |
| BnA05A4179-F | ATAATAAGTAATAGAGGAAGAC | 232 | 13549396 |
| BnA05A4179-R | ATACAACTAAGCCAACGT |  |  |
| BnA05A4188-F | TTCAATTCCAATGTAATAACCC | 184 | 13553638 |
| BnA05A4188-R | AGAAACGAAAAGCACCGA |  |  |
| BnA05A4205-F | CAAAGTCTCCATCCCACA | 204 | 13561051 |
| BnA05A4205-R | TAAATCCCAACCAATCAC |  |  |
| BnA05A4217-F | TTATAGGATTGATGGCGGAATA | 221 | 13563336 |
| BnA05A4217-R | CCAGCCCTTGCTTGCTTA |  |  |
| BnA05A4223-F | TCCCTCAGTATCCCGATGT | 227 | 13571406 |
| BnA05A4223-R | TGTCCTGTCCTCCCGTGT |  |  |
| BnA05A4226-F | ATGTTCATAAATACGTCCAC | 193 | 13571918 |
| BnA05A4226-R | TCCATCTCACTTTGCTTC |  |  |
| BnA05A4231-F | AGTAGAGTAGTAGCCAAAGTC | 291 | 13572075 |
| BnA05A4231-R | TATTTAGGTTAGCGGTTG |  |  |
| BnA05A4250-F | TTAATCAGATGGTACTCA | 231 | 13580208 |
| BnA05A4250-R | TTTTAGACGACTAACAAG |  |  |
| BnA05A4261-F | AGGAAGATTAGTTTTGTG | 292 | 13582761 |
| BnA05A4261-R | GTAAAATAAGAAAGCACA |  |  |
| BnA05A4266-F | AAAGCGATAGCCTAATGG | 190 | 13583600 |
| BnA05A4266-R | TAGCAGGTTTGTGAGCAAG |  |  |
| BnA05A4275-F | ATACATTGCTCGTCGTCC | 297 | 13585284 |
| BnA05A4275-R | CTCGCTTTGCTTCATCTC |  |  |
| BnA05A4293-F | TTGGTATTTGAATCGGTT | 241 | 13590398 |
| BnA05A4293-R | TACCCACAAGCAACATTC |  |  |
| BnA05A4297-F | GGGTTTAGTTTGGGAGGG | 198 | 13591806 |
| BnA05A4297-R | AGCGTTTTACCGGACAGA |  |  |
| BnA05A4299-F | CGGGTTTAGTTTGGGAGG | 199 | 13591856 |
| BnA05A4299-R | AGCGTTTTACCGGACAGA |  |  |
| BnA05A4318-F | TTGAGACTTATGCCCACC | 228 | 13594487 |
| BnA05A4318-R | TGAACCTGCCTCTTTGAC |  |  |
| BnA05A4329-F | GACCACGGTTTCCACGAT | 176 | 13597964 |
| BnA05A4329-R | TGTCAAGCCTAATGTCACTCA |  |  |
| BnA05A4330-F | CGGTTGTCCAAACTGTCA | 239 | 13597985 |
| BnA05A4330-R | ATGCTTCTGCTCCGAATG |  |  |
| BnA05A4331-F | CTGACAGCCCATTGAGAA | 182 | 13598327 |
| BnA05A4331-R | CAGGGACAACAAAGAAGC |  |  |
| BnA05A4332-F | TAGGGATGTATGCTTTTA | 197 | 13598733 |
| BnA05A4332-R | TCTACGGAGACTTACATTAT |  |  |
| BnA05A4333-F | TAGGGATGTATGCTTTTA | 197 | 13598751 |
| BnA05A4333-R | TCTACGGAGACTTACATTAT |  |  |
| BnA05A4341-F | CGTCAGCGTTTTCCCAGTT | 181 | 13599988 |
| BnA05A4341-R | CGGCGAGTTGAGTCATTATTTC |  |  |
| BnA05A4348-F | TATTGAGCCCAAGAAGAA | 190 | 13601293 |
| BnA05A4348-R | CGCTAGGGATATTACATT |  |  |
| BnA05A4373-F | GCAAAGGCGTGCTACATC | 298 | 13613313 |
| BnA05A4373-R | CTGGCTCTTCCTACTTACAT |  |  |
| BnA05A4385-F | ATCCTAAACTTAGCTTTA | 203 | 13619399 |
| BnA05A4385-R | GTTGCTTACTCTATTCAC |  |  |
| BnA05A4390-F | GCAATCCAAGCCATAAAG | 204 | 13620323 |
| BnA05A4390-R | AAAGGGCTCTGGTGCTAT |  |  |
| BnA05A4394-F | TCCATTGATTGGTGCTCA | 187 | 13621329 |
| BnA05A4394-R | ATTTTCAGGCTGCTTGTC |  |  |
| BnA05A1229-F | CCACATTCATAACGCCTAT | 196 | 13930615 |
| BnA05A1229-R | GCATCCCAGACAACAATC |  |  |
| BnA05A1279-F | ATTGGCACAGCCGTAAGA | 195 | 13939931 |
| BnA05A1279-R | GCGTTCCAAATCCGAGAC |  |  |
| BnA05A1330-F | TCTGCCATAACCCTTCGT | 231 | 13953313 |
| BnA05A1330-R | CCTACTTTACCCACAGATTG |  |  |
| BnA05A1371-F | TGCTAAACTGTAAAGGGAT | 207 | 13963203 |
| BnA05A1371-R | GATTGTGAAATTGGGATG |  |  |
| BnA05A1411-F | ATTAATCAAGAGACAAA | 217 | 13978305 |
| BnA05A1411-R | TGATAGGCTTTGTCTCTT |  |  |
| BnA05A6-F | CGACGAATCTTCTCAAAC | 283 | 13622507 |
| BnA05A6-R | AGCAAAGTCCAGCTACAA |  |  |
| BnA05A20-F | AAACTACGAGCATATGAA | 195 | 13625478 |
| BnA05A20-R | GATGATTTTGTTACCTGG |  |  |
| BnA05A39-F | AGTAGCAGTACAAGGCACA | 284 | 13631923 |
| BnA05A39-R | GATATTAACTAGGACGAAGGT |  |  |
| BnA05A45-F | CCTTGAAAATTAGCAGAA | 172 | 13633659 |
| BnA05A45-R | TGATGGTGGTGAATAGAG |  |  |
| BnA05A65-F | TCCTACGAGCAAACCCAG | 241 | 13637098 |
| BnA05A65-R | GAGTACAAATCGGAACCCT |  |  |
| BnA05A80-F | TGAAGAGTACCCAAGAAC | 194 | 13639835 |
| BnA05A80-R | ATAGAAGATGATGTAAAAGG |  |  |
| BnA05A86-F | TCAGCAGAATAGCGGTTAC | 226 | 13640788 |
| BnA05A86-R | TCTCAAAGAGGTCCTTACATT |  |  |
| BnA05A111-F | CCTCGTCTCAGTCGTGTC | 181 | 13650074 |
| BnA05A111-R | ACCAGTCGCTTAGTTATCAT |  |  |
| BnA05A144-F | TTGTTTTCGTTTGTGAGT | 190 | 13659400 |
| BnA05A144-R | TACCGTTATTTATTGGTG |  |  |
| BnA05A152-F | AGTGATACGGTAGGAAAC | 247 | 13661057 |
| BnA05A152-R | TCATTAGAAGGGAACAAA |  |  |
| BnA05A160-F | ATGATGACGTGAGAAAGCAC | 239 | 13662184 |
| BnA05A160-R | AACCCACTAGCATTAAGTAAAA |  |  |
| BnA05A176-F | GGATGCTACCCGAACAAA | 266 | 13665905 |
| BnA05A176-R | TATGGCTAAACTTCTCCTGAAT |  |  |
| BnA05A309-F | CCAGAAGAATGACCAGCC | 179 | 13699164 |
| BnA05A309-R | ACCAGACCAGTAACTCCG |  |  |
| BnA05A347-F | TATTTCCTCTTGGCTGTC | 218 | 13709791 |
| BnA05A347-R | GCCTCCCATTATCTCCTA |  |  |
| BnA05A460-F | AGTTGAGACTGTTTCCCA | 214 | 13730876 |
| BnA05A460-R | TTTCCTTCATCTTTGTTG |  |  |
| BnA05A498-F | GAAACAGAAAGCCAGGAG | 281 | 13738909 |
| BnA05A498-R | TGACTTGGTGACAGGGAT |  |  |
| BnA05A610-F | CCTGCTAGATCAATCCATACT | 227 | 13770341 |
| BnA05A610-R | CATAACCCAAACCTCCCT |  |  |
| BnA05A652-F | ACGGTGAGTTTCGTTCTT | 247 | 13779998 |
| BnA05A652-R | CAGGCATCATTCGTTTCT |  |  |
| BnA05A751-F | AGTAACGCTTTGAATGGT | 261 | 13808020 |
| BnA05A751-R | TTTGGATGAATATGGAGA |  |  |
| BnA05A845-F | AGCTTACAACCCAACAAA | 195 | 13839444 |
| BnA05A845-R | TGATGAAGGCGTGAATGA |  |  |
| BnA05A906-F | GGACATTCATCCCGTTTC | 190 | 13849786 |
| BnA05A906-R | ATCTTTGTGGTGGGTTCG |  |  |
| BnA05A957-F | CTTGCGCTGAAGGTAATG | 224 | 13860344 |
| BnA05A957-R | TGTGGGTGTTGGTGTTTG |  |  |
| BnA05A1067-F | TGGAGACGATGATGGGTT | 196 | 13887141 |
| BnA05A1067-R | GAATGTGCTATTTGTGGC |  |  |
| BnA05A1124-F | TGATGAACTTGATGGGAATG | 184 | 13905450 |
| BnA05A1124-R | GTGCCGTCTCAGATAGGC |  |  |
| BnA05A1206-F | GGTTGTGCCGTCTATGTG | 174 | 13925904 |
| BnA05A1206-R | CTTAGGGTAATTTGAGTGGTAT |  |  |
| BnA05A1227-F | ATTCTCGTGCTCAATCAGTC | 177 | 13929921 |
| BnA05A1227-R | GATCTACGCCAACAAAGG |  |  |
| BnA05A1353-F | AGCAAGATTCCTCAAGAC | 193 | 13958974 |
| BnA05A1353-R | TAATGGGTAATACACGGT |  |  |
| BnA05A1408-F | GTCTCCCGCCTATTGGTT | 281 | 13977723 |
| BnA05A1408-R | GGCTCGGTTAGCCTTGGT |  |  |
| BnA05A1507-F | AGCTTCTTCACCCTCCCC | 278 | 14004661 |
| BnA05A1507-R | ATCCACCTCAGCCAAAAT |  |  |
| BnA05A1539-F | AGTAGAATAATGGGGTCG | 187 | 14012099 |
| BnA05A1539-R | TAAACTGCTTGTCTTGGA |  |  |
| BnA05A1659-F | CCTTGTTTGTACTTGATGA | 183 | 14091401 |
| BnA05A1659-R | ATTGTATGCCTTGGTGAG |  |  |
