# Supplementary material for: Fine mapping of an up-curling leaf locus (BnUC1) in Brassica napus
Source: BMC Plant Biol. 2019 Jul 19;19:324. doi: 10.1186/s12870-019-1938-0 (PMC6642557; doi:10.1186/s12870-019-1938-0)
Supplement: Supplementary file 6 — Table S3. The designed qRT-PCR primers used in this study. (DOCX 14 kb) [file 12870_2019_1938_MOESM6_ESM.docx]

**Additional file 6: Table S3** The designed qRT-PCR primers used in this study.

| Name of Primers | Sequence of primers | Product length |
| --- | --- | --- |
| BnA05g240RT-F | ATTGACGAGGATGATTTGC | 151 bp |
| BnA05g240RT-R | TTTGACAGTGAAGGAGGAA |  |
| BnA05g250RT-F | CGCTAACTACGACTACTTGCC | 118 bp |
| BnA05g250RT-R | CATCCCTGATACACCACCACT |  |
| BnA05g260RT-F | CATCTTCAACAGAACCCTC | 169 bp |
| BnA05g260RT-R | CAGACGCAAGTCTCCTAA |  |
| BnA05g270RT-F | GTTGCAGCAGAGCAATAC | 162 bp |
| BnA05g270RT-R | AAATCAGCCAGTCCAGAG |  |
| BnA05g280RT-F | CCAAAGCACAGGCAATCA | 190 bp |
| BnA05g280RT-R | CAAGAGGAGAAGGGCAAG |  |
| BnA05g290RT-F | ATTACTGGCGTTAGCACTT | 124 bp |
| BnA05g290RT-R | TATTCCTTTCGACGGATC |  |
| BnBDG-RT-F  BnBDG-RT-R | CATCCTCCACAAGACTCACA  AACAGTAGCAGGGTTTCCA | 168 bp |
| BnFDH-RT-F  BnFDH-RT-R | TCTTCGGTGTCTTTGTCTTG  CGTCTCATCGCCTATTCCT | 215 bp |
| BnActin-RT-F | ATTCAGCCCCTTGTTTGTG | 147 bp |
| BnActin-RT-R | GTAAGCGTCTTTTTGACCCAT |  |
